# Malignant spinal cord compression: a retrospective audit of clinical practice at a UK regional cancer centre

**DOI:** 10.1038/sj.bjc.6602957

**Published:** 2006-01-24

**Authors:** A McLinton, C Hutchison

**Affiliations:** 1 Beatson Oncology Centre, Western Infirmary, Dumbarton Road, Glasgow G11 6NT, UK; 2 North Glasgow University Hospitals NHS Division, Western Infirmary, Dumbarton Road, Glasgow G11 6NT, UK

**Keywords:** malignant spinal cord compression, audit

## Abstract

Malignant Spinal Cord Compression (MSCC) is a particularly challenging area of cancer care where early diagnosis and expert multiprofessional care and rehabilitation, are paramount in optimising quality of life. This audit reports data collected retrospectively over a period of 12 months on patients with MSCC referred to the West of Scotland Cancer Centre (*n*=174). It was carried out to build on the work of the Clinical Resource and Audit Group (CRAG) and to examine current practice for symptom assessment, multiprofessional care and rehabilitation of patients with MSCC admitted to the cancer centre. Areas of concern include poor assessment of pain, the poor ambulatory status of patients on admission and the lack of clear plans for mobilisation and rehabilitation for the majority of patients. Recommendations include the development of regional guidelines for referral, treatment and rehabilitation, and the development of a pathway of care for use in all care settings across the region, together with improvements for use in patient information, staff education, audit and research. These are now being taken forward through the West of Scotland Cancer Network with dedicated funding from Macmillan Cancer Relief.

Malignant spinal cord compression (MSCC) is a common oncological emergency that requires prompt recognition and immediate treatment to relieve pain and preserve neurological function (Bucholtz, 1999). It can present at any time during the natural history of a cancer (Bucholtz, 1999) and is a major cause of morbidity in oncology patients (Husband *et al*, 2001). The effects of MSCC can range from minor sensory, motor and autonomic changes to severe pain and complete paralysis that significantly affects a patient's quality of life (Held and Peahota, 1993). In patients with back pain and a history of cancer, a high degree of suspicion is necessary to ensure an early diagnosis (Held and Peahota, 1993).

Much has been written about the management of patients with MSCC emphasising the importance of early diagnosis in maximising response to treatment and optimising patient outcomes (Held and Peahota, 1993; Bucholtz, 1999; Quinn and DeAngelis, 2000). In addition, the importance of a multidisciplinary approach to care is considered to be of vital importance in the rehabilitation of patients with MSCC (Held and Peahota, 1993; Hillier and Wee, 1997; Guo *et al*, 2003).

A national audit carried out in Scotland by the Clinical Resource and Audit Group (CRAG) over 16 months from January 1998 to April 1999, reported variations in care and suboptimal practices at many stages of the patient journey both prior to and following a diagnosis of MSCC (Levack *et al*, 2001, 2002). Despite these findings and the resulting recommendations, patients continue to present with advanced symptoms of MSCC and to experience inappropriate variations in care. While Levack *et al* (2001) did much to raise the profile of such a challenging area of cancer care and provided helpful recommendations to address the shortfalls, it is questionable whether these recommendations have been followed and whether there has been any improvement in local time to diagnosis, assessment of patient symptoms and treatment delivery. The impetus for this audit came from the Research and Practice Development Group (RPDG) within the cancer centre, to build on the work of Levack *et al* (2001) and to examine current practice for symptom assessment, multi-professional care and rehabilitation of patients with MSCC admitted to the cancer centre.

## PATIENTS AND METHODS

### Sample

The study sample included patients with a diagnosis of MSCC admitted to the cancer centre for treatment, over a 12-month period from November 2001 to October 2002. These patients were initially identified through liaison with the coding department and included all patients coded with a diagnosis of spinal cord compression or cauda equine syndrome. Patients who were admitted more than once relating to the same spinal cord episode, with either deterioration of their physical condition or for other symptom management, were included in the audit for the acute MSCC event only. A potential sample of 202 patients was initially generated. The final sample number included in the audit was 174 patients, with a further 17 patients included for details on primary cancer diagnoses (*n*=191).

Patients were excluded from the audit if:

- They were coded as MSCC but on further review of the case-notes did not have such a diagnosis although it had been suspected (*n*=11).
- It was not possible to obtain their case-notes within the timeframe of the audit due to difficulty with access (*n*=17).

### Data collection and analysis

In consultation with key multiprofessional experts a questionnaire was developed by the RPDG. Specific audit categories were identified from review of the literature, discussion and reflections on clinical practice. The audit questionnaire was piloted on 20 patient case records and refinements made to the design and content prior to the development of an electronic data collection tool which was created using Epi-Info™, 2003. Data was extracted from the written documentation of all professional groups involved in the care of these patients. This was analysed using the Epi-Info™ (2003) package and included basic descriptive statistics.

## RESULTS

### Diagnosis

The commonest primary tumour diagnoses were lung (29%), prostate (19%) and breast (13%). When combined, these comprised 61% (117/191) of all cases of MSCC. A relatively high incidence of MSCC, was also associated with either an unknown primary site (14%) or haematological malignancies (10%). The remaining 15% comprised of a further nine primary cancer diagnoses.

### Sites of compression

The thoracic spine was the most common site involved with 77% of patients being affected. A further 29% occurred in the lumbar region, 12% in the cervical region and 7% in the sacral region. Twenty-five percent of patients were identified as having MSCC in more than one area of the spine.

### Source of referral

**Figure 1:**
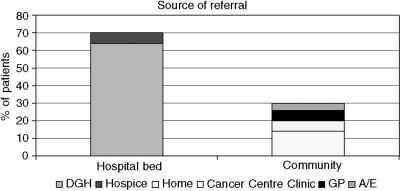
Source of referral to the cancer centre.

Patients were admitted to the cancer centre from a wide variety of health care settings and routes throughout the region. These can be split into two categories; those coming from the hospital setting (DGH and hospice) and those coming direct to the cancer centre from the community (GP, A/E, home and cancer centre clinic) as shown in Figure 1. As would be expected, the majority of patients (94%) were admitted to the cancer centre as an emergency.

### Magnetic resonance imaging/computerised tomography scanning

All patients had a magnetic resonance imaging (MRI) or computerised tomography (CT) scan to confirm diagnosis with the exception of one patient. In all, 58% had an MRI scan following admission to the cancer centre, while 36% had an MRI scan and 6% had a CT scan prior to being referred. The time interval between scanning and admission to the cancer centre is shown in Table 1. The majority of patients (67%) had their scan performed within 1 day either side of admission.

### Treatment

The majority of patients (93%) received palliative radiotherapy; 4% were treated with chemotherapy; 3% received surgery to treat their compression and 4% received supportive care alone, specific to presenting clinical symptoms. A small number of patients (4%) were treated with more than one treatment modality.

Although only 3% of patients received surgery to treat their compression the actual number referred for a surgical opinion following admission to the cancer centre was 7%.

Of the patients who received radiotherapy, 93% had one area of the spine treated and the remaining 7% had treatment to either two or three sites. Table 2 demonstrates the time interval between the MRI or CT scan being carried out and radiotherapy commencing. Most patients (61%) began radiotherapy either the same day or within 1 day of the MRI scan.

### Steroid therapy

From examination of medical and nursing letters from transferring hospitals and referring GP's, it was possible to identify if steroids had been prescribed prior to admission to the cancer centre. In 42% of cases, dexamethasone had been prescribed prior to cancer centre admission and this increased to 96% following admission. A dose of 16 mg per day was prescribed for 95% of the sample.

### Mobility

**Figure 2:**
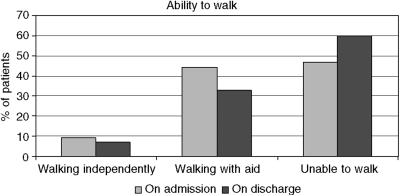
Ability to walk on cancer centre admission and discharge.

The ambulatory status of patients on admission and discharge is shown in Figure 2. At time of admission, only 9% of patients were able to walk independently without aid, 44% with aid of a walking stick, walking frame or assistance of another individual and 47% were unable to walk. Of the 159 patients who were discharged from the cancer centre, 7% were walking independently, 33% were walking with aid and 60% were unable to walk.

From the case records it was identified that on admission to the cancer centre, the majority of patients (86%) were initially nursed in a supine position on ‘bed rest’ and 3% remained ambulant. For 11% it was not possible to ascertain from either nursing or medical notes if restrictions were imposed regarding mobility. A plan of incremental movement was documented for 33%; the content of this varied from no plan to a structured plan located in the physiotherapy notes. Case records were examined to establish whether preventative measures had been taken against the development of thrombosis due to immobility. Antiembolic stockings were worn by 67% of patients and prophylactic anticoagulant therapy was prescribed for 35%.

### Pain assessment

Nearly all patients (98%) had a degree of pain at time of admission to the cancer centre. The site of pain was documented for 73% of patients with 25% having no site of pain mentioned and 2% documented to have ‘no pain’. A pain score was reported for 37% of patients with 24% having this reassessed following initial assessment.

### Bladder and bowel function

A total of 66% of patients’ required urinary catheterisation either for the management of urinary incontinence or urinary retention. In all, 32% had a catheter inserted prior to admission to the cancer centre and 34% following admission. There was documented evidence in the case-notes suggesting symptoms of constipation for 60% of patients on admission.

### Referral to physiotherapist and occupational therapist

The majority of patients (94%) were referred to the physiotherapy team with most referrals made within one day of admission (60%). In contrast, 18% were referred to occupational therapy, with the majority (55%) being referred ⩾7 days following hospital admission.

### Discharge location

The majority of patients (60%) were referred back to a DGH on discharge from the cancer centre. A further 17% were discharged to a hospice and the remaining 23% were discharged home.

### Survival/outcomes

Survival data was collected from case records and via the local electronic case management system. This identified that 15 patients (8.6%) died during their admission period in the cancer centre. Within 3 months (90 days) of admission, 74% of the audit population were known to be deceased.

## DISCUSSION

Results have been reported in relation to a variety of aspects of clinical care, as well as demographic and referral patterns for patients with MSCC. Much of the data is consistent with that reported in the literature, however, evidence is lacking in relation to some areas of practice and this is reflected in the results which will now be discussed.

Diagnoses and sites of compression for patients in this audit are consistent with findings from the prospective audit conducted by the Clinical Resource and Audit Group (CRAG) in Scotland (Levack *et al*, 2001) and a literature review by Loblaw and Laperriere (1998). Patients with lung, prostate and breast cancer had a higher incidence of MSCC, with the thoracic spine being the most common site affected.

MRI scanning of the entire spine is the investigation of choice in patients with known malignancy and suspected spinal canal disease (Cook *et al*, 1998; Loughrey *et al*, 2000; Husband *et al*, 2001; Levack *et al*, 2001). Less than half of the patients in this study had an MRI or CT scan prior to admission to the cancer centre. Levack *et al* (2001) similarly, found substantial delays from time of presenting symptoms to definitive radiological diagnosis with an MRI or CT scan. However, it is acknowledged that access to MRI and CT imaging is mainly confined to larger healthcare settings and that clinical demand often exceeds availability, resulting in some delay even when the scan is urgent. Further work is required to determine where specific delays are encountered and where improvements can be made.

Urgent referral and treatment of MSCC is essential to maximise prognosis and treatment outcomes (Markman, 1999). Our data shows that two thirds of patients received their scan within 24 h either side of admission to the cancer centre, which indicates the urgency with which these patients were referred for diagnosis.

It is suggested that initial presentation direct to a cancer centre is associated with reduced delay in time to treatment and improved neurological function (Husband, 1998). Husband (1998) strongly advises that patients who have back pain and a diagnosis of cancer be referred urgently or to be encouraged to self refer to the cancer centre. Several authors have advocated that the rate of early diagnosis and prevention of potential paralysis could be improved if oncology patients were taught the importance of contacting health care providers with complaints of pain, especially when the pain is accompanied by neurological signs and symptoms (Peterson, 1993; Husband, 1998; Bucholtz, 1999). Currently, there is no system in place within the cancer centre, to inform or educate patients on the early warning signs of MSCC that would alert them to present earlier. Since MSCC occurs in only a small percentage of patients it could be considered reasonable to target patient education to those at high risk, for example those with breast, lung and prostate cancer with bone metastases (Husband, 1998).

Palliative radiotherapy was the treatment of choice for 94% of patients presenting with MSCC, which is consistent with published literature (Quinn and DeAngelis, 2000). Very few patients were referred for surgical opinion (7%) from the cancer centre with even fewer undergoing surgery (3%). While this audit did not detect patients referred directly to surgery from a DGH for surgery alone it will have captured patients referred in this manner, who received adjuvant radiotherapy postsurgery.

The low rate of surgical referral could be due to unclear referral pathways to orthopaedic and neurosurgical expertise. This needs to be addressed particularly in light of the Patchell *et al* (2003) study, which showed that patients who were randomised to receive surgery (within 24 h of presentation) followed by radiotherapy (within 14 days) retained the ability to walk significantly longer than those treated with radiotherapy alone. Length of survival was not significantly different between the two groups, although there was a trend towards longer survival time in the surgery group. Those treated with surgery and radiotherapy walked for almost all their remaining life while those treated with radiotherapy alone were nonambulatory.

The time interval between MRI/CT scan and radiotherapy commencing was calculable for 156 of the 163 patients who received radiotherapy. In 63% of patients, radiotherapy was commenced on either the same day or within 1 day of the MRI scan. However, 22 patients did not commence radiotherapy until 5 days or more after their MRI/CT scan being performed. Data was not collected on reasons for delays to treatment and this needs further investigation in order to eliminate avoidable causes.

Steroids are an effective adjunct to radiotherapy, reducing spinal cord oedema and inflammation and improving neurologic deficits and pain control (Held and Peahota, 1993; Maranzano *et al*, 1996; Bucholtz, 1999; Quinn and DeAngelis, 2000). Based on patients’ signs and symptoms and a high index of suspicion for MSCC, steroid administration should be initiated prior to the completion of all necessary diagnostic tests (Bucholtz, 1999; Quinn and DeAngelis, 2000). Less than half of the patients appeared to have been prescribed dexamethasone prior to admission to the cancer centre. However, documentation received from referring DGHs and community sources did not always include information on the commencement of steroid therapy. Steroid therapy could have been initiated earlier in some patients prior to diagnosis, however, fair evidence exists suggesting that steroids do not have to be given routinely where a patient has good motor function at time of presentation, (Maranzano *et al*, 1996). It is common practice in the UK for 16 mg of Dexamethasone to be prescribed per day for MSCC (Levack *et al*, 2001). Ninety-five percent of patients in this audit received this dose.

Ambulatory status on admission was very similar to figures reported for this cancer centre in the audit by Levack *et al* (2001). This demonstrates that after four and a half years (time between data collection for the two audits), patients continue to be referred to the cancer centre with advanced symptoms of MSCC. It was also disappointing to see that ambulatory status of the group was generally worse on discharge with more patients being unable to walk. In some cases this could be the result of delays in time to diagnosis and referral for treatment, leading to poorer treatment outcomes. It is acknowledged, however, that data in relation to ambulatory status on discharge should be interpreted with caution, since it was difficult to obtain an objective picture from documentation in the case notes. Mobility was often poorly documented and assessed differently by different health care professionals.

It is standard practice for patients with MSCC at the cancer centre to be placed on bed rest, in a supine position, until stability of the patients’ spine is established and the treatment plan instigated. It was apparent from the audit that in the majority of cases there was no clear plan for individual patient mobilisation or guidance regarding positioning. This may have resulted in some patients lying supine for unnecessary and indefinite periods of time. A plan for incrementally increasing movement was found in the case records for only one third of patients. There appears to be a vacuum of evidence in the literature regarding appropriate positioning and optimal programmes for incremental movement in patients with MSCC. Jacobs (1999) suggests that some patients such as those who have a short life expectancy and those who have been paralysed for more than 24 h, would derive greater benefit from sitting up and reducing their risk of complications from bed rest. Further research is desperately needed to clarify practice in this area and to establish the true cost in terms of the complications associated with enforced bed-rest (Jacobs, 1999).

It is recognised that thrombosis is a common complication of malignancy (Arkel, 2000) and that patients with spinal cord injury are at risk of deep venous thrombosis (Chiou-Tan *et al*, 2003). The Scottish Intercollegiate Guidelines Network (SIGN) in their National Clinical Guideline – Prophylaxis of Venous Thromboembolism (VTE) (SIGN 62) recommends that all patients admitted to hospital with an acute medical illness that is likely to require bed rest for 3 days or more, should be individually assessed for risk of VTE (SIGN 62, 2002). Many patients with MSCC fall into this category. In the absence of any contraindications, leg exercises and the wearing of above knee graduated elastic compression stockings (GECS) are recommended as VTE prophylaxis (SIGN 62, 2002). While the audit findings suggest antiembolic stockings to be the most common measure used for the prevention of thrombosis development, it was not documented why one third of patients did not have these fitted. While 35% of patients had prophylactic anticoagulant therapy prescribed, further research is necessary to determine the role of prophylactic anticoagulant therapy in patients with MSCC.

Nearly all patients had pain at the time of admission to the cancer centre, with 3/4 having the location documented. SIGN, in their National Clinical Guideline – Control of Pain in Patients with Cancer – advocate that prior to the treatment of pain, an accurate assessment should be performed to determine the type and severity of pain and it's effect on the patient (SIGN, No 44, 2000). Disappointingly, despite a pain assessment tool being available, just over one third of patients had their pain assessed and a pain score documented. Even fewer had this pain reassessed during hospitalisation. This does not suggest that patients were not having their pain managed but it does question whether this was being carried out systematically or effectively.

Bowel and bladder incontinence develop with advanced autonomic nervous system involvement and carries a poor prognosis (Held and Peahota, 1993). Two thirds of patients required a urinary catheter for the management of urinary incontinence and retention. This is substantially higher than the 36% reported by Levack *et al* (2001). This highlights the late stage of presentation and the need for earlier diagnosis and treatment, before bladder function is affected. It is not surprising that so many patients (67%) in this audit were found to have symptoms of constipation on admission, as this is a common problem in patients with spinal cord compression or cauda equina syndrome (Fallon and O’Neill, 1997).

Effective rehabilitation is an important part of the management of spinal cord compression secondary to advanced malignancy and requires the diverse skills of all members of the multidisciplinary team (Hillier and Wee, 1997). Referral should be made when the patient is initially diagnosed with MSCC (Guo *et al*, 2003). Most patients were referred for physiotherapy assessment, which was usually carried out within 1 day of admission. It was surprising to find that so few patients were referred to occupational therapy and that in the majority of cases, referral was made at least 7 days after admission. It is not clear why so few patients were referred and why referrals were made so late. An awareness of the service and importance of referral is needed to reverse this trend. Referral criteria and guidelines may assist this process.

A large number of patients were discharged back to their local DGH following treatment in the cancer centre with patients also discharged back to hospice and home care settings. This emphasises the need for MSCC management to be consistent in all care settings across the region to ensure appropriate rehabilitation and symptom control.

A total of 74% of the audit population died within 3 months of their hospital admission, which reflects the poor prognosis of this patient group. If patients with MSCC are to have improved quality of life and extended survival, it is without doubt that current delays in the patient journey require to be tackled in order to improve this statistic.

While the audit has provided useful data in relation to referral, treatment and management of MSCC, it must be acknowledged that data was collected retrospectively and in some cases was incomplete. It relies on accuracy of documentation and therefore, in some cases may not provide a complete account of the care provided.

### Recommendations

This audit has identified important areas for practice development, research and education for the whole multiprofessional team in relation to the management of patients with MSCC and beyond. Many areas that have been highlighted may not be unique to this particular cancer centre and lessons can be learned to improve practice and processes in other care settings where patients with MSCC are treated.

Recommendations are summarised below:

- Development and implementation of local and regional guidelines for the referral, management and rehabilitation of patients with suspected or diagnosed MSCC. This should be done as a network-commissioned piece of work involving all stakeholders and full consultation, based on existing evidence, best practice and taking into account local provision and access to resources. Guidelines would need to be introduced with an extensive education programme to raise awareness and optimise future compliance.
- Development and implementation of a pathway of care for multiprofessional use across the region, to support the guidelines, and promote consistency and equity of care, regardless of care setting. This would also help to facilitate communication internally within the cancer centre and externally with both primary and secondary care.
- Development of local and regional education programmes to correct deficits in knowledge and clinical practice. The information needs of stakeholders such as GP's and general physicians will also require to be addressed if appropriate referral to the cancer centre is to be achieved. This could be done by developing a core presentation covering the key issues and linking it to existing programmes such as the educational aspect of the Gold Standards Framework (Thomas, 2003) in primary care.
- Patient information:
  ○ Development of MSCC-specific patient information in collaboration with national voluntary agencies such as CancerBACUP to be given to patients with a diagnosis of MSCC.
  ○ Improvement in consistency of cancer site-specific information in relation to MSCC for high-risk cancer groups, which could be done through Cancer Managed Clinical Networks.
- Research strategy to address gaps in evidence such as;
  ○ Ambulation/rehabilitation and rationale of nursing patients in a supine position, the duration of this and the cost in terms of complications.
  ○ Effect on time to diagnosis/ambulatory status/outcomes of direct referral to a cancer centre.
  ○ DVT prophylaxis in patients with MSCC.
- Establishment of a core data set in collaboration with ISD for future prospective audit.

These recommendations are now being taken forward through the Regional Cancer Network as an 18-month project with dedicated funding from Macmillan Cancer Relief.

## CONCLUSIONS

Care of patients with MSCC is complex and involves multiprofessional expertise. Several recommendations have been presented around the areas of guideline development, documentation, multiprofessional education, patient information, research and audit which if addressed, will help to ensure that patients with MSCC receive appropriate quality care and management that is based on available evidence and best practice.

## Figures and Tables

**Table 1:** Time interval between MRI/CT scan and cancer centre admission

**MRI/CT scan**	**%**
>1 day prior to admission	11
1 day prior to admission	28
Same day as admission	24
1 day after admission	15
2 days after admission	5
3 days after admission	2
4 days after admission	2
>4 days after admission	10
Unknown data	3
	
Total	100

**Table 2:** Time interval between MRI/CT scan and radiotherapy commencing

**Radiotherapy commenced**	**%**
Prior to scan	1.5
Same day as scan	25
1 day after scan	36
2 days after scan	15
3 days after scan	4
4 days after scan	0.5
>4 days after scan	13
Unknown data	4
	
Total	100
